# Deep learning-based transcriptome model predicts survival of T-cell acute lymphoblastic leukemia

**DOI:** 10.3389/fonc.2022.1057153

**Published:** 2022-11-02

**Authors:** Lenghe Zhang, Lijuan Zhou, Yulian Wang, Chao Li, Pengjun Liao, Liye Zhong, Suxia Geng, Peilong Lai, Xin Du, Jianyu Weng

**Affiliations:** ^1^ The Second School of Clinical Medicine, Southern Medical University, Guangzhou, China; ^2^ Department of Hematology, Guangdong Provincial People’s Hospital, Guangdong Academy of Medical Sciences, Guangzhou, China

**Keywords:** T-cell acute lymphoblastic leukemia (T-ALL), survival, transcriptome sequencing, deep learning, k-means

## Abstract

Identifying subgroups of T-cell acute lymphoblastic leukemia (T-ALL) with poor survival will significantly influence patient treatment options and improve patient survival expectations. Current efforts to predict T-ALL survival expectations in multiple patient cohorts are lacking. A deep learning (DL)-based model was developed to determine the prognostic staging of T-ALL patients. We used transcriptome sequencing data from TARGET to build a DL-based survival model using 265 T-ALL patients. We found that patients could be divided into two subgroups (K0 and K1) with significant difference (P< 0.0001) in survival rate. The more malignant subgroup was significantly associated with some tumor-related signaling pathways, such as PI3K-Akt, cGMP-PKG and TGF-beta signaling pathway. DL-based model showed good performance in a cohort of patients from our clinical center (P = 0.0248). T-ALL patients survival was successfully predicted using a DL-based model, and we hope to apply it to clinical practice in the future.

## 1 Introduction

It is well known that T-cell acute lymphoblastic leukemia (T-ALL) is a blood disease with high clinical incidence. The pathogenesis of T-ALL is complex. Genetic factors, viral infection and some toxic compounds can promote the occurrence of T-ALL (1). About a quarter of adult leukemia, and 15% of childhood leukemia are T-ALL (2). With the promotion of combined chemotherapy, the therapeutic effect of T-ALL has been significantly improved. However, there is a large difference in survival among different patient cohorts. Still 50% of adults patients die from T-ALL, compared to 20% in pediatric (3, 4). T-ALL has complex etiology and high heterogeneity among different patients, which makes prognosis prediction of T-ALL very difficult (5). The prognosis of patients will greatly affect the choice of treatment, so there is an urgent need to develop tools to predict patient survival (6).

The molecular subgroups of T-ALL have been extensively studied by researchers (7). In recent years, different T-ALL subgroups have been found to have unique gene expression signatures that reflect thymocyte development (8). Early T−lineage progenitor (ETP) leukemia often has adverse outcomes. However, T lymphocytic leukemia with CD1a^+^, CD4^+^, or CD8^+^ immunophenotype presents a relatively favorable prognosis (9). However, most studies do not take survival information into account when identifying subgroups (10). Instead, people tend to introduce survival information to observe the clinical significance of these subgroups (11). The result is that many subgroups do not show significant differences in survival time (12). In fact, the survival time of T-ALL subgroups need to be take into account at the beginning of exploration.

In order to address these issues, we use a deep learning (DL) framework on the T-ALL datasets. The deep learning framework we use is called autoencoder. Autoencoder has the function of representing learning algorithm in general sense. It has been shown that autoencoders can effectively generate prognostic features (13, 14). The high-dimensional nature of gene expression data often causes difficulties in analysis, but autoencoders have demonstrated their ability to cope with high-dimensional data (15, 16). It is worth noting that autoencoder spontaneously focuses on genes with similar pathways, so the use of autoencoders will facilitate the interpretation of biological functions (17). In this study, reliable molecular subgroups of T-ALL were obtained through comprehensive and accurate big data calculation, which could withstand the test of external cohorts.

We trained the model with 265 T-ALL samples from the Therapeutically Applicable Research to Generate Effective Treatments (TARGET) database. We found two subgroups with significant difference in survival time. These two subgroups have been identified as having independent predictive values for patient outcomes. Most importantly, the two subgroups derived from our DL framework have been successfully validated in self-built real-world patient cohorts. By analyzing these two subgroups, we found new genes and pathways that significantly affect the prognosis of T-ALL. As a result, this paper presents a significant DL-based model for predicting the prognosis of patients with T-ALL.

## 2 Methods

### 2.1 Data collection

In this study, 265 transcriptome sequencing samples from patients with T-ALL were collected from the TARGET database (https://ocg.cancer.gov/programs/target). After the removal of 21 samples with unknown survival status and 3 samples with unclear survival time, 241 T-ALL samples were finally included in the study. In order to minimize batch effects between data from different sources and to allow the model to be applied to a larger scale, the fragments per kilobase of transcript per million fragments mapped (FPKM) expression values were used in this study. To make the final model more explanatory, all gene IDs were converted to official gene symbols based on Gencode v22 (www.gencodegenes.org).

At the same time, we selected 20 T-ALL patients from the Hematology Department of Guangdong Provincial People’s Hospital for follow-up, and performed transcriptome sequencing of their bone marrow mononuclear cells at the time of initial diagnosis. There were 15 male patients and 5 female patients. The 20 patients had an average age of 33.8 at diagnosis, ranging from 18 to 90 years. The study was approved by the Ethics Committee of Guangdong Provincial People’s Hospital. The approval Number is 2019463H(R1). The data was available at the GEO under accession numbers GSE214998.

### 2.2 Total RNA extraction

The lymphocytes separation medium (TBDscience, China) was used for the isolation of mononuclear cells from bone marrow aspirate. The Trizol reagents were added (Invitrogen, USA) to the sample and let stand for 10 minutes. Then chloroform was added, mixed well and let stand for 3 minutes. After centrifugation at 12,000 × g for 15 minutes at 4°C, the supernatant was taken and mixed with isopropyl alcohol of equal volume and let stand for 10 minutes. The supernatant was removed after centrifugation at 12,000 × g for 10 minutes at 4°C, and the precipitate was washed with ethanol. After centrifugation at 7,500 × g for 5 minutes at 4°C, the supernatant was removed and allowed to stand and dry for 15 minutes. Mix with DNase for 30 minutes and wash once with ethanol. Finally, RNase-free DDW was used to dissolve the RNA. Monitoring RNA contamination and degradation was carried out using AGAR gels. The NanoPhotometer spectrophotometer (IMPLEN, USA) was used to determine the purity of RNA, while the Bioanalyzer 2100 system (Agilent Technologies, USA) was used to determine the integrity of RNA.

### 2.3 mRNA library construction

mRNA was purified using magnetic beads and Oligo(dT)-attached magnetic beads. Libraries were generated using the NEBNext UltraTM RNA Library Prep Kit for Illumina (NEB, USA). In NEBNext First Strand Synthesis Reaction Buffer, divalent cations were used for fragmentation at high temperatures. In order to synthesize first strand cDNA, M-MuLV reverse transcription enzyme was used in conjunction with random hexamer primers. Using RNase H and DNA Polymerase I to synthesis the second strand cDNA. For hybridization, the NEBNext adaptor with hairpin loop structure were ligated. 250~300 bp cDNA fragments were selected by AMPure XP system (Beckman Coulter, USA). Before polymerase chain reaction (PCR), we used USER enzyme (NEB, USA) to digest cDNA at 37°C for 15 min followed by 95°C for 5 min. The PCR is performed using Phusion High-Fidelity DNA polymerase, Universal PCR primers, and Index (X) Primer. The assay results were evaluated using Agilent Bioanalyzer 2100 system (Agilent Technologies, USA). The PCR products were purified using Beckman Coulter’s AMPure XP system.

### 2.4 Transcriptome sequencing and quality control

The TruSeq PE Cluster Kit (Illumia) was used to perform the clustering of the index-coded samples. Sequencing was performed on an Illumina Novaseq platform, with 150-bp paired-end reads. From raw fastq data, we removed reads containing ploy-N or low quality reads, as well as reads containing adapters. Clean Data was used for subsequent experiments.

### 2.5 Deep learning framework

Autoencoder (AE) is a kind of Artificial Neural Networks (ANNs) used in semi-supervised learning and unsupervised learning, its function is to learn the representation of input information (18). The structure of autoencoder is divided into encoder and decoder. There are two main characteristics of autoencoder: one is that the number of neurons in the input layer is equal to that in the output layer (reconstructed layer); the other is that there is a bottleneck layer in the network. Given the input space X∈χ and the feature space h∈ℱ, the autoencoder resolves the mapping *f* of the two, and *g* minimizes the reconstruction error of the input feature:


f:χ→ℱ

g:ℱ→χ

$$f,\;g=\arg\underset{f,g}{\min}{\left|\left|X-g\left[f\left(X\right)\right]\right|\right|}^{2}$$

After the solution is completed, h , the feature output by the encoder, can be regarded as a representation of the X . We build an autoencoder with 5 layers in Python (v3.8.12) based on TensorFlow 2.7.0 (https://github.com/tensorflow), including input layer, output layer and 3 hidden layers. The three hidden layers contain 500, 100 and 500 neurons respectively. The hidden layer in the middle becomes the bottleneck of the entire network, forcing the network to compress the input data and generate new features. The 50% dropout was used to make the entire network more robust. The whole network training process we carried out 10 epochs.

### 2.6 K-means clustering

From the autoencoder bottleneck layer, we got 100 new features of all the raw data. In order to identify features with log-rank P values less than 0.05, we performed a univariate Cox-PH model with these new features. Then we use these condensed features for k-means clustering algorithm from the sklearn Python package (19, 20). The Silhouette score (21) was used to determine the optimal value of K.

### 2.7 Data partitioning and robustness assessment

We artificially partitioned the TARGET database into training and test sets. The training set needs to have sufficient sample size, and the test set also needs to have as many samples as possible. In order to give consideration to both aspects, we finally chose 60/40% split. We wanted to use the cross-validation method to partition the dataset. The K-fold cross-validation algorithm from the sklearn Python package can quickly split 50/50%, 80/20%, or 90/10%. But to split 60/40%, we had to manually random partition the TARGET database into 5 folds, and then randomly select 3 of them as training set and the remaining 2 as test set. We did this 10 times and got 10 training/test sets. If the training/testing process is repeated using this data partitioning, the robustness of the model can be fully evaluated. Finally, all samples from TARGET database were used to train autoencoder and classifier to predict the labels of 20 T-ALL patients from the Hematology Department of Guangdong Provincial People’s Hospital.

### 2.8 Supervised classification

An eXtreme Gradient Boosting (XGB) algorithm (22) was trained from the sklearn Python package using the labels obtained by the K-means algorithm. Data standardization pipelining was based on the training set, using StandardScaler of the sklearn Python package (23). The standard value of sample X was calculated with the following function:


$$\text{Z}=\frac{\left(X-U\right)}{S}$$

where S is the variance of the training samples, and U is the mean of the training samples. When training the XGB models, we used K-fold cross-validation (k=5) and grid search from the sklearn Python package to find the optimal hyperparameters. The sample grouping probability output from the XGB model was used as the survival risk score.

### 2.9 Alternative approaches to the deep learning framework

In order to explore whether the Deep Learning framework could be replaced in this study, we tried two alternative approaches to carry out the experiment. The first alternative was to use principal components analysis (PCA) to reduce the dimension of the input data to replace the features generated by 100 neurons in the autoencoder bottleneck layer. We choose to retain at least 95% of the explained variance ratio. The second alternative was to use single-variant Cox-PH models to reduce the number of features in the input data. After the completion of the two alternative approaches, K-means clustering was required to observe whether patients with significant differences in survival time could be grouped.

### 2.10 Functional analysis

We performed some functional analysis to reveal the potential factors that could significantly affect the survival time of T-ALL patients.

#### 2.10.1 Clinical covariate analysis

We collected and compared the clinical covariates of patients in K0 and K1. Including the age of patients at diagnosis, peripheral blood white blood cell (WBC) count, peripheral blood lactate dehydrogenase (LDH), bone marrow blasts percentage and WT1 expression in bone marrow cells measured by PCR. As well as whether the patient underwent hematopoietic stem cell transplantation (HSCT).

#### 2.10.2 Differential expression

To identify differentially expressed genes between survival subgroups, differential expression analysis was performed on mRNA expression. We used the limma (v3.32.7) R package (24). The selection criteria for differentially expressed genes was adj.P< 0.05 and absolute fold change values ≥ 2.

#### 2.10.3 Enriched pathway analysis

By using clusterProfiler (v3.14.3) R package, we analyzed pathways enriched by up-regulated and down-regulated genes according to the Kyoto Encyclopedia of Genes and Genomes (KEGG). The inclusion criteria for pathways were P< 0.05, minimum count > 3, maximum count< 5000 and adj.P< 0.1.

#### 2.10.4 Tumor microenvironment analysis

The StromalScore, ImmuneScore and ESTIMATEScore were calculated using ESTIMATE (25). CIBERSORTx (https://cibersortx.stanford.edu/) was used to analyze infiltrating immune cells in 22 tumors.

## 3 Results

### 3.1 Two differential survival subgroups are identified

**Figure 1:**
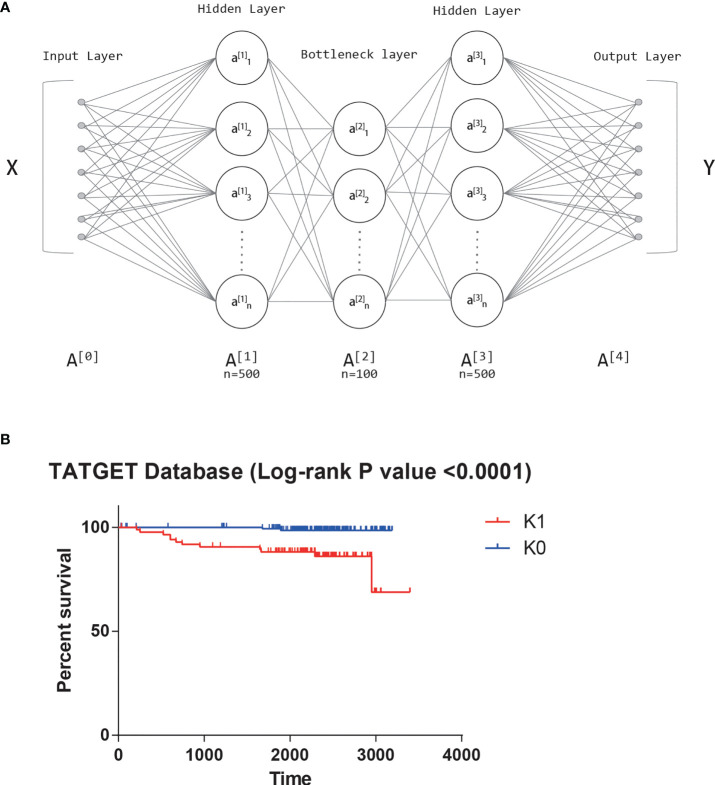
**(A)** The structure diagram of autoencoder. **(B)** Significant survival difference of datasets from TARGET database.

From TARGET database, we obtained the transcriptome sequencing data of 241 T-ALL patients, and each patient had mRNA expression levels of 24991 genes. All features were first input into the autoencoder (18). An autoencoder structure diagram can be seen in **Figure 1A**. As new features, we used the weight of 100 neurons in the bottleneck layer.

Univariate Cox-PH model was performed on 100 new features, and 42 features that were correlated with survival (log-rank p-value<0.05) were selected. K-means clustering determined 42 features subjectively, and the value of K was tried from 2 to 10. The Silhouette score was calculated after each clustering, and the score closest to 1 was selected. Finally, the best K value was 2. That is, the optimal cluster number was 2. The two groups obtained by clustering were called K0 and K1. There was also a significant difference in survival between the two clusters based on the KM survival curves (log-rank P value<0.0001, **Figure 1B**).

**Figure 2:**
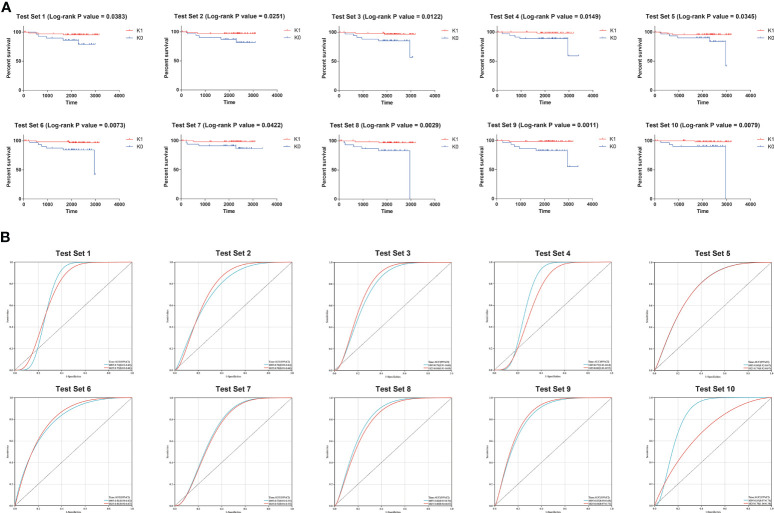
**(A)** Results of the 10 times KM curves for XGB model. **(B)** Results of the 10 times time-dependent ROC curve for XGB model.

Therefore, K0 and K1 were used as labels of XGB classifier for supervised learning in subsequent training. The samples from the TARGET database were randomly split 10 times into 60% and 40%. We used the KM survival curve and the time-dependent ROC curve to evaluate the accuracy of XGB model predictions (**Figure 2**). The mean log-rank P value of KM survival curve was 0.01864. The mean area under a receiver-operating characteristic curve (AUC) for 3-year overall survival of time-dependent ROC curve was 0.789, and the mean AUC for 5-year overall survival was 0.766. These results suggested that XGB classifier could robustly distinguish individuals from different survival subgroups.

### 3.2 Independent cohorts are robustly validated for survival subgroups

**Figure 3:**
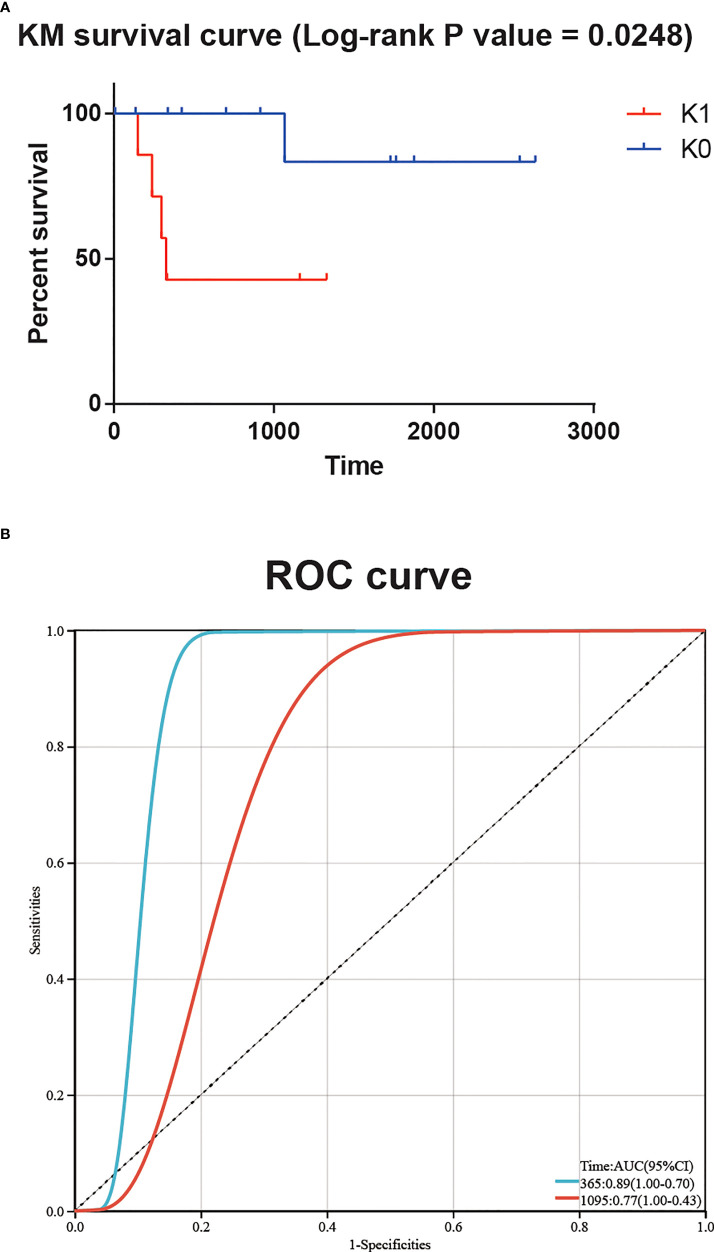
**(A)** Significant survival difference for 20 T-ALL patients from Guangdong Provincial People’s Hospital. **(B)** The time-dependent ROC curve showed a significant correlation between patient risk score and survival time.

In order to verify the robustness of the classifier and its application prospect in actual clinical work, we used the transcriptome sequencing data of 20 T-ALL patients from Guangdong Provincial People’s Hospital as an independent cohort input model. These 20 patients were divided into K0 group and K1 group, and the KM survival curve showed a significant difference in survival between the two groups (log-rank P value = 0.0248, **Figure 3A**). The time-dependent ROC curve also showed a significant correlation between patient risk score and survival time (AUC for 1-year overall survival = 0.89, AUC for 3-year overall survival = 0.77, **Figure 3B**).

### 3.3 The deep learning-based methodology exceeds alternative approaches

**Figure 4:**
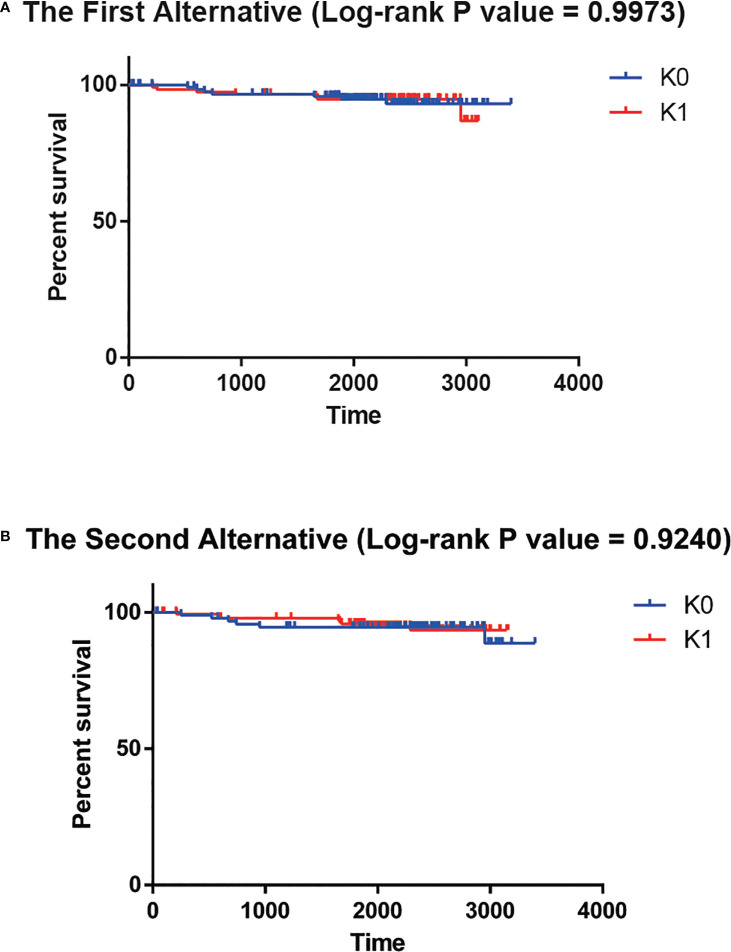
**(A)** No significant survival difference for the first alternative. **(B)** No significant survival difference for the second alternative.

To explore the need for a Deep Learning framework, we used two possible alternatives. The first alternative was to use PCA to reduce the dimension of the input data instead of the features generated the autoencoder bottleneck layer. While preserving at least 95% of the explained variance ratio, we reduced the input data to 182 dimensions. Then univariate Cox-PH model was performed to screen out the parts of the 182 principal components that were obviously correlated with survival time (log-rank p-value<0.05). That left 9 principal components. However, there was no significant difference in survival time between K0 group and K1 group (log-rank P value = 0.9973, **Figure 4A**). The second alternative was to use the single-variant Cox-PH model to reduce the number of features in the input data. We obtained 359 features that were significantly associated with survival time (log-rank p-value<0.05). Similarly, there was no significant difference in survival time between K0 group and K1 group (log-rank P value = 0.9240, **Figure 4B**). These results indicated that neither of the alternatives could achieve the effect of the Deep Learning framework.

### 3.4 Clinical correlates of survival subgroups

We compared the associations of clinical covariates that were previously thought to have a possible effect on patient survival with subgroups in this study (**Table 1**). We did not observe clearly statistically significant indicators. K0 and K1 subgroups may be an independent predictor of T-ALL prognosis.

**Table 1:** Associations of survival subgroups with clinical covariates.

Variable	Overall, N = 20	K0, N = 12	K1, N = 8	P-value*
**Gender**				0.3
** Female**	5 (25%)	2 (17%)	3 (38%)	
** Male**	15 (75%)	10 (83%)	5 (62%)	
**Age**	28 (24, 32)	28 (24, 32)	28 (22, 39)	>0.9
**WBC (×10^9/L)**	30 (5, 57)	36 (9, 57)	15 (4, 61)	0.4
**LDH (U/L)**	352 (220, 558)	352 (220, 725)	411 (236, 520)	>0.9
**WT1 (10^4)**	106 (45, 979)	265 (60, 979)	104 (38, 1,011)	>0.9
**HSCT**				>0.9
** Yes**	11 (55%)	7 (58%)	4 (50%)	
** No**	9 (45%)	5 (42%)	4 (50%)	
**Bone marrow blasts percentage**	90 (74, 91)	87 (74, 90)	90 (78, 91)	0.7

*Fisher’s exact test.

### 3.5 Functional analysis of the survival subgroups in TARGET database samples

**Figure 5:**
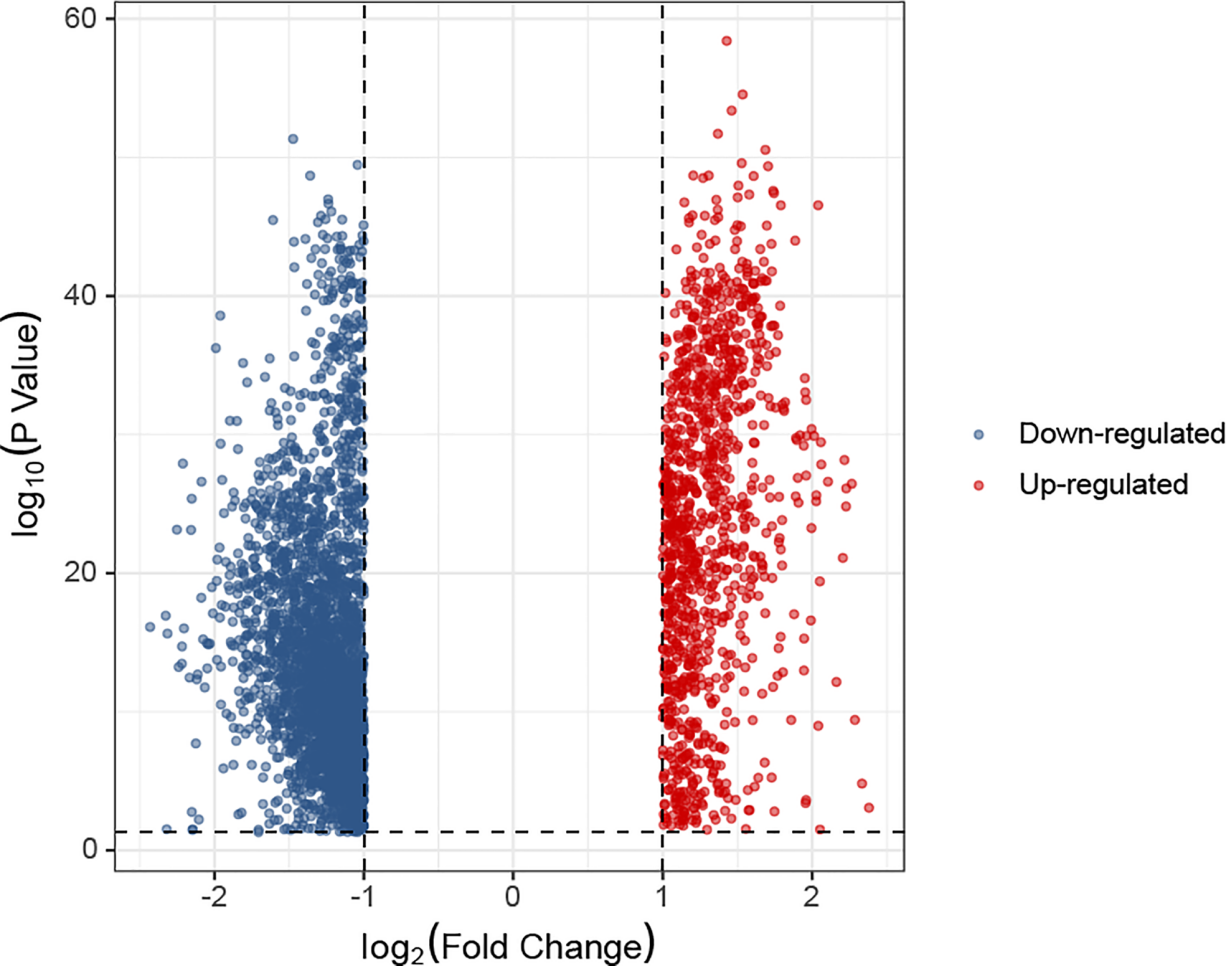
Difference in gene expression between K1 and K0 subgroups represented by a volcano plot.

In the K0 and K1 groups, differentially expressed genes were identified. The selection criteria for differentially expressed genes was adj.P< 0.05 and absolute fold change value ≥ 1. A total of 1085 up-regulated genes and 2957 down-regulated genes were screened from K1 group compared with K0 group. The comparative expression profile of these genes were shown in **Figure 5**. There were many leukemia-related genes in the up-regulated genes of K1 group, such as UBB (P=1.56e-50) (26), MIF (P=2.76e-50) (27), DRAP1 (P=1.72e-49) (28), RPS25 (P=1.9e-49) (29), EIF3K (P=4.77e-49) (30), SSNA1 (P=1.27e-48) (31), GPX4 (P=3.01e-48) (32), PHB2 (P=7.13e-48) (33), NME2 (P=2.44e-47) (34) or CFL1 (P=4.07e-47) (35).

**Figure 6:**
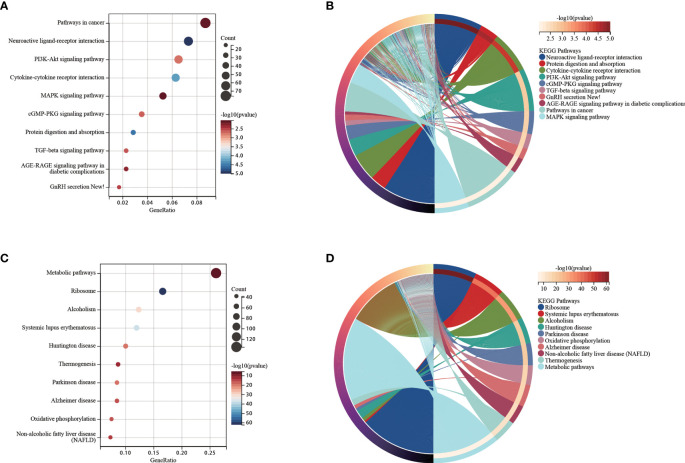
**(A)** Functional analysis for up-regulated genes in the K1 subgroup. **(B)** Chord diagram of enriched terms for up-regulated genes in the K1 subgroup. **(C)** Functional analysis for up-regulated genes in the K0 subgroup. **(D)** Chord diagram of enriched terms for up-regulated genes in the K0 subgroup.

We used functional analysis to explain the difference in the survival subgroups K0 and K1. Functional analysis of up-regulated genes in K1 subgroup were shown in **Figures 6A, B**. We observed that some cancer-related pathways were enriched in the K1 subgroup. PI3K-Akt pathway has increased activity in a large number of malignant tumors and promotes the growth of leukemia stem cells (36). It has been shown that the cGMP-PKG signaling pathway promotes leukemia cell proliferation (37). TGF-beta signaling pathway is a double-acting regulator that has been reported to benefit the immune escape of leukemia cells (38). GnRH signaling pathway is inhibited by Leukemia inhibitory factor (LIF) (39). AGE-RAGE signaling pathway strongly induces the proliferation of leukemia cells and cell lines (40). MAPK signaling pathway promotes drug resistance of leukemia cells (41). Functional analysis of the up-regulated genes in K0 subgroup were shown in **Figures 6C, D**.

### 3.6 Association between K-means survival subgroups and tumor microenvironment

**Figure 7:**
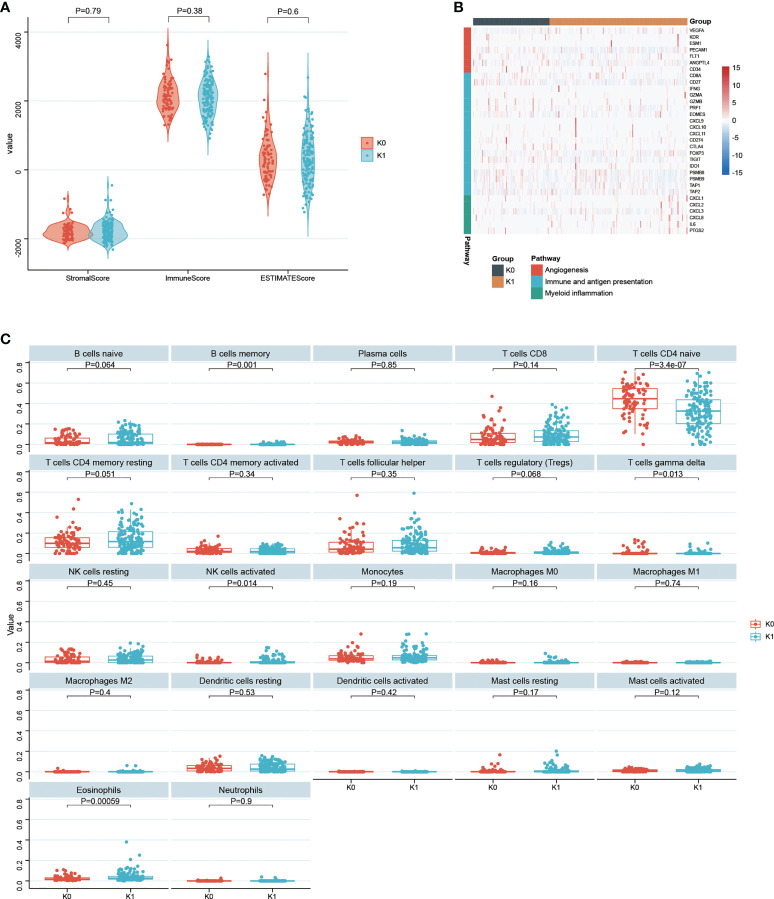
**(A)** Statistical comparison of StromalScore, ImmuneScore and ESTIMATEScore between K0 and K1 subgroups. **(B)** Gene expression in tumor microenvironmental pathways associated with immune and antigen presentation, angiogenesis, and myeloid inflammation. **(C)** 22 infiltrated immune cells between the K0 and K1 subgroups were analyzed.

There were no statistically significant difference between the StromalScore, ImmuneScore and ESTIMATEScore between the K0 and K1 subgroups (**Figure 7A**). There were no significant difference in immune function and antigen presentation, angiogenesis, and myeloid inflammation signaling pathways related to tumor microenvironment between K0 and K1 groups (**Figure 7B**). In the K1 group, B cells memory, NK cells activated, and eosinophils were significantly enriched by the CIBERSORT algorithm analysis (P< 0.05, **Figure 6C**). The K0 group was significantly enriched in T cells CD4 naive and T cells gamma delta (P< 0.05, **Figure 7C**).

## 4 Discussion

The heterogeneity of T-ALL has restricted people’s understanding of its etiology. We have seen many studies on novel typing of T-ALL. However, there are few types that can clearly predict the long-term survival status of patients. More importantly, most reported T-ALL subgroups have no validating using either external validation cohorts or external validation cohorts downloaded from a common database. Our study used data from patients we met in the real world to validate the model, which undoubtedly makes it more convincing. According to our knowledge, we are the first to use deep learning framework to construct a T-ALL prognostic prediction model. Our study will promote the application of deep learning framework in the development of prognostic prediction models. In practical application scenarios, the model can not only be used for prediction, but more importantly, clinicians can adjust the treatment plan of patients according to the prediction results.

We used a deep learning framework and identified two new subgroups of T-ALL based on transcriptome sequencing data. We verify the robustness of the model in many aspects. With the CV approach in particular, we can obtain continuous and repeatable good results. Presumably, deep learning frameworks have extracted information from high-dimensional data that is significantly related to survival time. But PCA and the like are clearly not. Somewhat unfortunately, the information extracted by deep learning frameworks cannot be intuitively understood by humans.

We observed that the K1 subgroup had a worse survival expectation, and in the functional enrichment analysis of the K1 subgroup, we did obtain pathways that have been reported to increase the severity of leukemia malignancy. K1 group members exhibited significantly higher levels of B cells memory, NK cells activated, and eosinophils according to the CIBERSORT algorithm analysis. It was also found that the K0 group is significantly enriched in CD4 naive and gamma delta T cells. This model has improved our understanding of the etiology of malignant T-ALL.

There are a few points that need to be discussed separately about our self-built external validation cohort. The first point is that the diagnosis time span of these patients is relatively long. The quality of recent patient samples is certainly better preserved, but the preservation of patient samples from many years ago (the earliest collection in 2015) could be very inconsistent. The second point is that there are differences in the way patients’ samples are stored. Most of the samples were dissolved in DDW in the form of RNA and refrigerated at minus 80 degrees Celsius. Some samples were directly refrigerated after total RNA was dissolved in Trizol. The third point is that patients’ clinical information is kept inconsistently. The loss of information may be due to frequent changes in the hospital’s record system in recent years, or it may be due to omissions in patient information collection years ago. Despite these problems, we obtained positive results in our external validation cohort, which demonstrated the robustness of this model. As clinicians and researchers, we look forward to conducting prospective studies in the future, not only to improve the model, but also to improve treatment for our patients.

## Data availability statement

The datasets presented in this study can be found in online repositories. The names of the repository/repositories and accession number(s) can be found below: https://www.ncbi.nlm.nih.gov/, GSE214998.

## Ethics statement

The studies involving human participants were reviewed and approved by ethics committee of Guangdong Provincial People’s Hospital. The patients/participants provided their written informed consent to participate in this study.

## Author contributions

LHZ, PLL, XD, and JYW conceived and designed this study. LHZ, LJZ, CL, YLW, PJL, LYZ, SXG, and PLL performed data analysis. LHZ, LJZ, XD, and JYW wrote the manuscript. All authors contributed to the article and approved the submitted version.

## Funding

This work was supported by the National Key R&D Program of China (No. 2017YFE0131600), the National Natural Science Foundation of China (Nos.81870121, 82070176, 82270161), the Natural Science Foundation of Guangdong Province, China (Nos. 2019B020236004, 2019B151502006, 2021A1515011436) and High-level Hospital Construction Project of Guangdong Provincial People’s Hospital (DFJHBF202107).

## Conflict of interest

The authors declare that the research was conducted in the absence of any commercial or financial relationships that could be construed as a potential conflict of interest.

## Publisher’s note

All claims expressed in this article are solely those of the authors and do not necessarily represent those of their affiliated organizations, or those of the publisher, the editors and the reviewers. Any product that may be evaluated in this article, or claim that may be made by its manufacturer, is not guaranteed or endorsed by the publisher.
